# Level of Depression and Associated Risk Factors Among the Elderly People Residing in Old Age Home in Bangladesh: A Cross‐Sectional Study Conducted in 2025

**DOI:** 10.1002/hsr2.72531

**Published:** 2026-05-17

**Authors:** Mehedi Hasan Naim, Mimma Tabassum, Md. Iftakhar Parvej

**Affiliations:** ^1^ Department of Statistics Noakhali Science and Technology University Noakhali Bangladesh

**Keywords:** aged, Bangladesh, depression, Geriatric Depression Scale (GSD), old age home (OAH)

## Abstract

**Background and Aims:**

Depression is now a worldwide public health issue among elderly, with particularly high prevalence among those residing in old‐age homes. In Bangladesh, limited research has been conducted on this vulnerable population. This study aimed to determine the prevalence of depression and identify associated factors among elderly individuals living in old‐age homes in Bangladesh.

**Methods:**

A cross‐sectional study was conducted from September 22 to November 15, 2025, among 158 elderly individuals ( ≥ 60 years) residing in old‐age homes in Gazipur, Chattogram, and Noakhali. Data on sociodemographic, lifestyle, social support, health, and institutional factors were collected, and depression was assessed using the short form of the Geriatric Depression Scale (GDS). Descriptive statistics summarized the data, while Chi‐square tests and binary logistic regression were used to identify associations and predictors of geriatric depression.

**Results:**

The study found that 82.3% of the elderly residents were experiencing depression and it increases with advancing age, particularly among those aged 70 years and above. Gender, type of illness, and satisfaction with services in old age homes were identified as significant predictors of depression among elderly residents. Females were considerably more likely to experience depression than males, and individuals with physical illnesses had higher odds of depression compared to those with mental health conditions. Moreover, participants reporting neutral satisfaction with old age home facilities were more prone to depression than those who were very satisfied.

**Conclusion:**

The findings indicate a high prevalence of depression among elderly residents of old‐age homes in Bangladesh, with several sociodemographic health‐related and institutional factors contributing to its severity. These results highlight the need for targeted mental health interventions and improved care services, which may assist policymakers in developing effective strategies to address geriatric mental health issues in Bangladesh.

## 1 Introduction

Globally, aging population is growing exponentially. The WHO projects that the percentage of older people will double between 2015 and 2050, where the majority residing in countries with low to moderate income levels. Health and social care systems face a lot of difficulties because of this demographic transition, including increased prevalence of physical disabilities, chronic diseases, and mental health conditions, including depression [1, 2]. Depression is a medical condition indicated by a loss of self‐worth, hopelessness, and a continuous sense of loneliness. A lower energy consumption and focus, problem with sleep (insomnia), hunger reduction, weight loss, and muscular pain are some of its side effects [3]. Particularly among elderly people, depression is prevalent. Elderly people of Bangladesh have different types of physical, emotional, and social health problems. Elder depression has been the subject of several research worldwide. Depression in elderly people is now become a global issue, yet the prevalence varies considerably by country. In the Western world, around 19.5% of older persons are affected by depression [4], but greater rates are observed in Sri Lanka (27.8%) [5], Pakistan (23%) [6], and Iran, where it can reach up to 58% [7]. In comparison, the prevalence is lower in Singapore (13.3%) [8] and Hong Kong (12.5%) [9]. In China, around 30.8% of older persons report depression [10], whereas rural Nepal has an even higher prevalence of 37.5% [11]. South Korea has rates ranging from 20% to 34% [12], in Japan, it is between 19.8% and 33.5% [13], while Taiwan has around 20.1% of its urban elderly people negatively affected [14]. When we focused on adults 60 or above 60 years, the appearance of depression varies significantly within the countries. India has the highest prevalence, with more than one in every four older persons (27.1%) suffering from it. Mexico follows closely with 23.7%, suggesting that nearly a quarter of the elderly in the country suffer from depression. In Russia, the average rate is reported to be 15.6%, whereas in Ghana it is lower at 11%. South Africa has an even lower number, with only 6.4% of its older population having depression. On the contrary, China has the lowest prevalence within this age group, with only 2.6% of older individuals reporting being depressed [15]. Besides geographic variations, it is also found that women are more depressed than men.

According to the first national study on mental health, conducted between 2003 and 2005, 16.1% of individuals had some form of mental disease. It also found from various studies that women were apparently more sufferers from a mental condition, with 19% affected compared to 12.9% of men [16]. According to previous studies done in Bangladesh, the frequency of mental diseases in adults varies between 6.5% and 31.0%, depending on the healthcare scenario or community continuity, and females tend to be at more risk [17].

Women, physical sickness and injury, illness and loss of function, memory loss, challenging life events, poor socioeconomic position, loneliness, social detachment, and less support are the main factors for depression in the elderly [18, 19, 20, 21, 22, 23]. According to WHO, factors that indicate elderly people at risk for depression include a history of chronic disease or physical pain, difficulty with challenges in daily living activities, behavioral characteristics (subservient, worriedness, or avoiding), unfavorable life instances (divorce, detachment, death, poverty, and communal separation), and an insufficient support of society [24].

Old age homes (OAHs) are institutional settings that provide accommodation and supportive services to elderly individuals who can no longer live independently or lack sufficient family care. Their prominence is increasing worldwide due to population aging, longer life expectancy, and shifting family structures, leading to greater demand for long‐term residential care alongside home‐ and community‐based services [25, 26]. Residential care models vary globally, with differences in quality, regulation, and services across high‐ and low‐income regions, reflecting broader cultural and socioeconomic contexts of aging populations [27].

Old‐age homes in Bangladesh are emerging as an important form of institutional elderly care due to rapid population aging and changing family structures. Traditionally, elderly care was provided within joint families; however, this system is gradually declining, increasing the need for residential care facilities [28, 29]. Institutional care for older adults is becoming a new social reality, often driven by factors such as family neglect, urbanization, and lack of support systems [30]. Despite growing demand, the development of old‐age homes and formal elderly care services remains limited and under‐regulated in Bangladesh [31, 32].

Depression among elderly residents of OAHs in Bangladesh remains under‐researched despite the growing aging population [33]. Different sociodemographic variables, along with elderly age, gender, educational qualification status, religious status, family type, residency, poverty, and occupation, have been linked to depression in many different studies [17, 34]. This study aims to assess the prevalence of depression and examine its association with sociodemographic, lifestyle, and psychosocial factors in this population. Specifically, it addresses which factors significantly influence depression among institutionalized older adults. The findings are intended to inform targeted interventions and policies to enhance mental health and well‐being in these settings.

## 2 Materials and Methods

### 2.1 Study Design and Participants

This study employed a cross‐sectional design and was conducted between September 22, 2025, and November 15, 2025. Data were collected through face‐to‐face interviews using a structured questionnaire among elderly individuals residing in old‐age homes in Gazipur, Chattogram, and Noakhali. A convenience non‐probability sampling technique was utilized for participant recruitment. This approach facilitated direct interaction with participants, enabling a comprehensive assessment of their living conditions, lifestyle, experiences, and associated factors.

All participants were informed about the purpose of the study prior to the interview, and the survey was conducted after obtaining their verbal informed consent. To ensure confidentiality and anonymity, no personal information was asked for in the survey, and all data was coded.

### 2.2 Data Collection Tool

After preliminary review of relevant literature, a draft questionnaire comprising both open‐ and close‐ended questions was developed, the majority of which were drawn from previous similar studies [35, 36, 37, 38, 39, 40, 41, 42]. The draft self‐administered questionnaire was bilingual (Bangla and English) and contained two sections. Sociodemographic, lifestyle and social support, health‐related, and institutional (OAH) variables were included in Section A and to evaluate the depression status within the older people who were staying in OAHs, we used Geriatric Depression Scale short form (GDS‐15) [43] in Section B.

### 2.3 Inclusion and Exclusion Criteria

Participants, aged 60 years or above, who agreed to take part in the study and had resided in OAHs for at least 6 months, were included. Those who were mentally sick or unstable (Dementia and Alzheimer disease), unable to clearly communicate, had any psychological disorder, or unwilling to participate, were excluded from the study.

### 2.4 Outcome Variable

Depression was the outcome variable of this study. The GDS‐15 is a useful measurement tool for assessing depression in older people living in OAHs or the community. A basic, user‐friendly tool to assess depression in elderly individuals, the Geriatric Depression Scale (GDS) was developed. The initial Geriatric Depression Scale was a 30‐item questionnaire that allowed elderly people to select Yes/No responses about their emotional state in respect of the past week [44]. Subsequently, 15 items from the original long form of the GDS were included in a short form of the GDS‐15, that showed the strongest correlation with depression in validation tests [45]. Some of the questions are negatively scaled, those indicated depression, when they replied “No.” On the other hand, 10 of the 15 items showed depression when replied positively (Yes). The participant took 5–7 min to complete the study.

Initially, the sum score of geriatric depression is calculated as the total value of the 15 depressing factors [43]. The resulting GDS Score is then interpreted as follows: a score ranging from 0 to 5 indicates a normal condition, whereas a score greater than 5 indicates depression.

The internal consistency of the Geriatric Depression Scale‐15 (GDS‐15) was evaluated using Cronbach's alpha. The original scale reported a Cronbach's alpha of 0.80 [46], while in the current study, the Cronbach's alpha was 0.799, indicating acceptable reliability for the study sample.

### 2.5 Independent Variables

This study considered four categories of independent variables: sociodemographic, lifestyle and social support–related, health‐related, and institutional (OAH)‐related factors. Sociodemographic variables included region (Noakhali, Chattogram, and Gazipur), age (60–70, 70–80, 80–90, and above 90), gender (men and women), religion (open ended), occupation (open ended), educational qualification (no formal education, primary, secondary, higher secondary, graduate, and postgraduate), marital status (married, unmarried, widowed, divorced, and separated) [42], number of children (3 or fewer and more than 3), and children educational level (no formal education, primary, secondary, higher secondary, graduate, and postgraduate) [35].

Lifestyle and Social Support–related factors included physical activity (daily, weekly, monthly, rarely, and never), recreational activities (no and yes,) [41], social/communal involvement (no and yes), regular visitors (no and yes), emotional support from staff & mates (no and yes) [39], daily activities (doing nothing, kitchen/crafting, sleeping/socializing, and religious/reading/watching TV) [37], mobile use (no and yes), visit frequency (daily, weekly, monthly, rarely, and never), meet with family/friends (outside the OAHs) (daily, weekly, monthly, rarely, and never), and make new friend/relationships (no and yes) [40].

Health‐related factors included current sickness (no and yes), sickness type (physical illness and mental illness) incurable/chronic diseases (open‐ended) [36].

Institutional (OAH)‐related factors include length of residing in OAHs (6 months, 1–2 years, and over 2 years) [47], OAHs type (private and government), services & facilities satisfaction in OAH (dissatisfied, neutral, satisfied, and very satisfied), and lived in any OAHs before (no and yes) [38].

### 2.6 Sample Size Determination

The required sample size for this study was calculated using Cochran's formula [48]. Based on a previously reported prevalence of depression among the elderly of 62.8% [49], the estimated sample size needed to obtain a statistically representative sample was approximately 359, assuming a 95% confidence level and a 5% margin of error.

Since old‐age homes typically have a limited and finite number of residents, applying the finite population correction (FPC) was necessary to avoid overestimation of the sample size [50]. Considering a total population of 260 elderly residents in the selected homes and the initially calculated sample size of 359, the adjusted minimum required sample size was reduced to 151. To accommodate potential non‐response, 165 eligible participants were approached, of whom 158 consented and completed the survey.

### Ethics Approval

Ethical approval (Ref: NSTU/SCI/EC/2025/461) was obtained from the Ethical Review Committee of the Faculty of Science, Noakhali Science and Technology University, Bangladesh. Prior to participation, all individuals provided informed consent, indicating that they fully understood the purpose and procedures of the study.

### 2.7 Statistical Analysis

Statistical Package for the Social Sciences (SPSS), Version 25.0 (IBM) [51] was used for the analysis of the data. Descriptive statistics (frequencies and percentages) were used to describe the background characteristics. The Chi‐square test was used to identify the association between these factors and depression. A binary logistic regression was applied as a multivariate analysis to find the influence of the independent variable on geriatric depression. A two‐sided *p*‐value < 0.05 was regarded as indicative of statistical significance. To evaluate the suitability of the model used in the analysis, the Nagelkerke R² statistic was employed as a measure of goodness of fit. Multicollinearity among the predictor variables was assessed using the standard error (SE), where an SE value below 0.5 suggests the absence of multicollinearity. In this analysis, the observed SE (0.208) indicates no multicollinearity issue, while the Nagelkerke R² (*p*‐value = 0.678) confirms that the model is well‐fitted and appropriate for the research data.

### 2.8 Statistical Test

#### 2.8.1 Chi‐Square Test

The *χ*² (Chi‐square) test was employed to evaluate whether significant associations exist between categorical variables. In this study, the test was used to examine the relationship between depression levels and various risk factors among older adults residing in OAHs in Bangladesh.

$H_{0}$: There is no association between depression levels and the risk factors.

$H_{1}$: There is a significant association between depression levels and the risk factors.

The analysis was performed at a 5% level of significance. The Chi‐square statistic represents a measure of the discrepancy between the observed frequencies and the frequencies that would be expected if there were no association.

A *p*‐value < 0.05 was considered statistically significant. If the *p*‐value fell below this threshold, the null hypothesis (*H*₀) was rejected, indicating a statistically significant association between depression levels and the risk factors; otherwise, *H*₀ was not rejected.

#### 2.8.2 Binary Logistic Regression

Binary logistic regression is an appropriate statistical method when the dependent variable has two categorical outcomes. In this study, the dependent variable is the level of depression among older adults living in OAHs in Bangladesh, classified as depressed and normal (not depressed). Predictor variables were included in the model to estimate the probability of an older adult being depressed.

$H_{0}$: The predictor variables have no significant effect on the level of depression.

$H_{1}$: The predictor variables have a significant effect on the level of depression.

The analysis was performed at a 5% level of significance. The logistic regression model describes the relationship between the predictor variables and the log odds of being depressed rather than not depressed. In essence, it quantifies how changes in the predictor variables influence the likelihood of depression.

A *p*‐value less than 0.05 was considered statistically significant. Predictor variables with *p*‐values below this threshold were interpreted as having a significant association with depression, while those with *p*‐values above 0.05 were considered not significantly associated.

## 3 Results

### 3.1 Descriptive Statistics of All Variables

A total of 158 respondents participated in this study. In Table 1, we show the sociodemographic, lifestyle and social support, health, and institutional (OAH)‐related characteristics with their frequency and percentages, which may present the overall visual representation of this study.

**TABLE 1:** Sociodemographic, lifestyle and social support, health, and institutional (OAH)‐related characteristics of respondents.

Variables	Categories	Frequency (*n*)	Percent (%)
Region	Noakhali	6	3.8
	Chattogram	49	31
	Gazipur	103	65.2
Age	60–70	52	32.9
	70–80	55	34.8
	80–90	34	21.5
	Above 90	17	10.8
Gender	Men	32	20.3
	Women	126	79.7
Religion	Muslim	104	65.8
	Hindu	39	24.7
	Buddhist	3	1.9
	Christian	12	7.6
Occupation	Homemaker	39	24.7
	Day laborer	76	48.1
	Private job	10	6.3
	Self employed	12	7.6
	Governmental job	8	5.1
	Businessman	13	8.2
Educational qualification	No formal education	70	44.3
	Primary	51	32.3
	Secondary	10	6.3
	Higher secondary	9	5.7
	Graduate	13	8.2
	Postgraduate	5	3.2
Marital status	Married	67	42.4
	Unmarried	23	14.6
	Widowed	44	27.8
	Divorced	14	8.9
	Separated	10	6.3
Number of children	3 or fewer	79	50
	More than 3	79	50
Children educational level	No formal education	14	8.9
	Primary	46	29
	Secondary	17	10.8
	Higher secondary	11	7
	Graduate	62	39.2
	Postgraduate	8	5.1
Length of residing in OAHs	6 months	35	22.1
	1–2 years	54	34.2
	Over 2 years	69	43.7
Using mobile (personal)	No	126	79.7
	Yes	32	20.3
Any regular visitors (children/family members/anyone)	No	90	57
	Yes	68	43
Visit frequency	Daily	5	3.2
	Weekly	1	0.6
	Monthly	21	13.3
	Rarely	44	27.8
	Never	87	55.1
Meet with family/friends (outside the OAHs)	Daily	7	4.4
	Weekly	6	3.8
	Monthly	1	0.6
	Rarely	23	14.6
	Never	121	76.6
Current sickness	No	68	43
	Yes	90	57
Sickness type	Physical illness	85	53.8
	Mental illness	73	46.2
Incurable/chronic diseases	High blood pressure (hypertension)	16	10.1
	Anemia and malnutrition	18	11.4
	Diabetic	18	11.4
	Cancer	14	8.9
	Neurological problem	12	7.6
	Cardiovascular disease (CVD)	21	13.3
	Chronic kidney disease (CKD)	7	4.4
	Physical impairment	12	7.6
	Lungs problem	10	6.3
		30	19
Daily activities	Doing nothing	8	5.1
	Assisting in the kitchen, crafting, and try to create something new	50	31.6
	Sleeping, moving around, and spending time with others	74	46.8
	Engage in religion activity, read books or newspapers, write, and watch television.	26	16.5
Physical activity	Daily	74	46.8
	Weekly	37	23.4
	Monthly	1	0.6
	Rarely	39	24.7
	Never	7	4.5
Social/communal involvement	No	82	51.9
	Yes	76	48.1
Recreational activities	No	37	23.4
	Yes	121	76.6
Make new friends/relationships	No	58	36.7
	Yes	100	63.3
Emotional support from staff & mates	No	28	17.7
	Yes	130	82.3
OAHs type	Private	64	40.5
	Governmental	94	59.5
Services & facilities satisfaction in OAH	Dissatisfied	15	9.5
	Neutral	38	24
	Satisfied	51	32.3
	Very satisfied	54	34.2
Lived in any OAHs before	No	152	96.2
	Yes	6	3.8
	Total	*N* = 158	100

From Table 1 we can see that, 65.2% of the total were from Gazipur. Nearly the second largest group was from Chittagong (31%). Only 3.8% came from Noakhali, which is a relatively small figure. Age 70–80 years old (34.8%) was the largest age group and women made up the majority (79.7%). The majority were Muslim (65.8%), followed by Buddhists (1.9%), Hindus (24.7%), and Christians (7.6%). Almost half (48.1%) were day laborers and 44.3% of participants had no formal education. We found that among the elderly people of OAH, 14.6% were single, 27.8% were widowed, and 42.4% were married. Half of the participants had three or fewer children and 39.2% of the children had completed graduate‐level education. For more than 2 years, most of the people (43.7%) stayed in OAHs. only 20.3% owned a personal cell phone, whereas 43% reported frequent visits from close relatives. Most respondents (55.1%) reported that no one came to visit them, and 76.6% said that they do not meet with family and friends outside OAH's. Currently, more than half (57%) were ill, with 46.2% experiencing mental distress and 53.8% experiencing physical health problems. The most prevalent chronic conditions were infectious (Viral/Communicable Disease) disorders (19%), cardiovascular disease (13.3%), diabetes (11.4%), and anemia/malnutrition (11.4%). Sleeping, going for a walk, and talking with others were the most popular daily activities (46.8%). Almost half (46.8%) of respondents exercised daily, 51.9% were not involved in any social or community activities. Approximately 76.6% were involved in different types of recreational activities and 63.3% of the respondents were interested in making new friends. The majority of the respondents (82.3%) received emotional support from staff and fellow residents. Most (59.5%) lived in government OAH, additionally 66.5% expressed their happiness or reported being satisfied with the facilities, while almost all (96.2%) had never previously resided in any old‐age homes.

### 3.2 Prevalence of Depression and Its Scores

Approximately 82.3% of the older individuals who were residing in OAHs were in depression. Mean score of the depression was 8.94 (SD = 3.502) (Table 2).

**TABLE 2:** Frequency distribution of the respondents by characteristic of the study.

Level of depression	Total	Percent (%)
Normal	28	17.7
Depression	130	82.3
Total	158	100

### 3.3 Bivariate Association Between Depression and Other Factors

In Table 3, a bivariate association (Chi‐square ($\chi^{2}$)) between depression and sociodemographic, lifestyle and social support, health, and institutional (OAH)‐related variables have been described.

**TABLE 3:** Chi‐square analysis of key^a^ respondent characteristics and their association with depression level.

Variables	Categories	Normal	Depression	Total	Chi‐square ($\chi^{2}$)	*p*‐value
Region	Noakhali	1	5	6	3.817	0.15
	Chattogram	13	36	49		
	Gazipur	14	89	103		
	Total	28	130	158		
Age	60–70	14	38	52	9.218	0.03
	70–80	6	49	55		
	80–90	8	26	34		
	Above 90	0	17	17		
	Total	28	130	158		
Gender	Men	18	14	32	40.852	< 0.001
	Women	10	116	126		
	Total	28	130	158		
Religion	Muslim	16	88	104	5.536	0.14
	Hindu	8	31	39		
	Buddhist	2	1	3		
	Christian	2	10	12		
	Total	28	130	158		
Occupation	Housewife	1	38	39	17.284	0.004
	Day laborer	14	62	76		
	Private employee	3	7	10		
	Self‐employed	1	11	12		
	Government employee	3	5	8		
	Businessmen	6	7	13		
	Total	28	130	158		
Educational qualification	No formal education	11	59	70	4.25	0.51
	Primary	10	41	51		
	Secondary	0	10	10		
	Higher secondary	3	6	9		
	Graduate	3	10	13		
	Postgraduate	1	4	5		
	Total	28	130	158		
Marital status	Married	13	54	67	5.45	0.24
	Unmarried	7	16	23		
	Widowed	6	38	44		
	Divorced	2	12	14		
	Separated	0	10	10		
	Total	28	130	158		
Number of children	3 or fewer	14	65	79	0	0.99
	More than 3	14	65	79		
	Total	28	130	158		
Children educational level	No formal education	2	12	14	10.923	0.05
	Primary	3	43	46		
	Secondary	3	14	17		
	Higher secondary	3	8	11		
	Graduate	13	49	62		
	Postgraduate	4	4	8		
	Total	28	130	158		
Length of residing in OAHs	6 months	8	27	35	1.531	0.47
	1–2 years	7	47	54		
	Above 2 years	13	56	69		
	Total	28	130	158		
Using mobile (personal)	No	16	110	126	10.765	0.001
	Yes	12	20	32		
	Total	28	130	158		
Any regular visitors (children/family members/anyone)	No	13	77	90	1.54	0.22
	Yes	15	53	68		
	Total	28	130	158		
Visit frequency	Daily	1	4	5	6.916	0.14
	Weekly	1	0	1		
	Monthly	6	15	21		
	Rarely	7	37	44		
	Never	13	74	87		
	Total	28	130	158		
Meet with family/friends (outside the OAHs)	Daily	2	5	7	5.994	0.20
	Weekly	3	3	6		
	Monthly	0	1	1		
	Rarely	5	18	23		
	Never	18	103	121		
	Total	28	130	158		
Current sickness	No	17	51	68	4.337	0.04
	Yes	11	79	90		
	Total	28	130	158		
Sickness type	Physical illness	10	75	85	4.477	0.03
	Mental illness	18	55	73		
	Total	28	130	158		
Incurable/chronic diseases	High blood pressure (hypertension)	4	12	16	8.675	0.47
	Anemia and malnutrition	3	15	18		
	Diabetic	2	16	18		
	Cancer	1	13	14		
	Neurological disorder	1	11	12		
	Cardiovascular disease (CVD)	5	16	21		
	Chronic kidney disease (CKD)	1	6	7		
	Physical impairment	5	7	12		
	Lung problem	2	8	10		
	Viral/communicable disease	4	26	30		
	Total	28	130	158		
Daily activities	Doing nothing	4	4	8	10.869	0.01
	Assisting in the kitchen, crafting, and try to create something new	10	40	50		
	Sleeping, moving around, and spending time with others	7	67	74		
	Engage in religion activity, read books or newspapers, write, and watch television.	7	19	26		
	Total	28	130	158		
Physical activity	Daily	16	58	74	7.02	0.14
	Weekly	6	31	37		
	Monthly	1	0	1		
	Rarely	4	35	39		
	Never	1	6	7		
	Total	28	130	158		
Social/communal involvement	No	11	71	82	2.169	0.14
	Yes	17	59	76		
	Total	28	130	158		
Recreational activities	No	5	32	37	0.587	0.44
	Yes	23	98	121		
	Total	28	130	158		
Make new friends/relationships	No	6	52	58	3.42	0.06
	Yes	22	78	100		
	Total	28	130	158		
Emotional support from staff & mates	No	2	26	28	2.612	0.11
	Yes	26	104	130		
	Total	28	130	158		
OAHs type	Private	18	46	64	7.985	0.005
	Governmental	10	84	94		
	Total	28	130	158		
Services & facilities satisfaction in OAH	Dissatisfied	1	14	15	16.363	0.001
	Neutral	1	37	38		
	Satisfied	8	43	51		
	Very satisfied	18	36	54		
	Total	28	130	158		
Lived in any OAHs before	No	26	126	152	1.043	0.31
	Yes	2	4	6		
	Total	28	130	158		

^a^ Sociodemographic, lifestyle and social support, health, and institutional (OAH).

The Chi‐square test revealed significant associations between depression and several factors among the 158 elderly residents of old‐age homes. Depression was significantly associated with age ($\chi^{2}$ value = 9.218 and *p* = 0.03), gender ($\chi^{2}$ value = 40.852 and *p* < 0.001), occupation ($\chi^{2}$ value = 17.282 and *p* = 0.004), children education levels ($\chi^{2}$ value = 10.923 and *p* = 0.05), use of a personal mobile phone ($\chi^{2}$ value = 10.765 and *p* = 0.001), current sickness ($\chi^{2}$ value = 4.337 and *p* = 0.04), type of sickness ($\chi^{2}$ value = 4.477 and *p* = 0.03), daily activities ($\chi^{2}$ value = 10.869 and *p* = 0.01), type of OAH's ($\chi^{2}$ value = 7.985 and *p* = 0.005), and services & facilities satisfaction in OAH ($\chi^{2}$ value = 16.363 and *p* = 0.001) (Table 3). These findings indicate that depression among the residents is significantly associated with some of the sociodemographic, lifestyle and social support, health, and institutional (OAH)‐related variables.

The Chi‐square test indicated that region, religion, educational qualification, marital status, number of children, length of residing in OAHs, family visits, frequency of visitors, meet with family/friends (outside the OAHs), chronic diseases, physical activity, social/communal involvement, recreational activities, make new friendships, emotional support from staff & mates, and previous residence in OAHs were not significantly associated with depression (Table 3). This implies that sociodemographic and health‐related variables, along with institutional environment, may exert a substantial influence on the psychological well‐being of older adults, whereas certain sociodemographic, lifestyle and social support variables may not be significantly associated.

### 3.4 Depression With Respect to Associated Factors (Logistic Regression)

Here, a binary logistic regression approach was used to determine the factors associated with depression. In this model, GDS was treated as a binary dependent variable, and variables identified as significant in the bivariate analysis were included as predictors. The results of the logistic regression analysis are summarized in Table 4.

**TABLE 4:** Analysis of risk factors associated with depression (Binary Logistic Regression).

Variables	Category	*p*‐ value	Odds ratio (95% CI)
Age	60–70	.	Ref
	70–80	0.81	1.257 [0.188–8.415]
	80–90	0.35	0.324 [0.031–3.364]
	Above 90	0.99	4.74E8 [0.000]
Gender	Male	.	Ref
	Female	0.001	123.852 [8.080–1898.442]
Occupation	Housewife	.	Ref
	Daily laborer	0.51	0.395 [0.024–6.407]
	Private job holder	0.87	1.380 [0.028–67.596]
	Self‐employed	0.51	28.161 [0.000–1.05E7]
	Government job holder	0.97	1.077 [0.024–47.559]
	Businesses	0.92	1.197 [0.033–43.815]
Children educational level	No formal education	0.68	2.388 [0.036–158.536]
	Primary	0.13	19.220 [0.424–870.580]
	Secondary	0.32	8.074 [0.127–513.546]
	Higher secondary	0.57	2.841 [0.077–104.126]
	Graduate	0.76	1.761 [0.045–69.559]
	Postgraduate	.	Ref
Using mobile (personal)	No	.	Ref
	Yes	0.24	0.269 [0.030–2.413]
Current sickness	No	.	Ref
	Yes	0.32	0.411 [0.071–2.374]
Sickness type	Mental problem		Ref
	Physical problem	0.02	8.886 [1.334–59.197]
Services & facilities satisfaction in OAH	Dissatisfied	0.31	5.138 [0.212–124.828]
	Neutral	0.002	211.880 [7.464–6014.550]
	Satisfied	0.09	5.180 [0.768–34.959]
	Very satisfied	.	Ref
Daily activities	Doing nothing	.	Ref
	Assisting in the kitchen, crafting, and try to create something new	0.50	4.835 [0.050–469.880]
	Sleeping, moving around, and spending time with others	0.38	7.881 [0.077–801.955]
	Engage in religion activity, read books or newspapers, write, and watch television.	0.19	23.271 [0.220–2456.514]
OAHs type	Private	.	Ref
	Governmental	0.22	0.235 [0.023–2.364]

Table 4 shows significant association and explaining by odds ratio for the variables in terms of older individuals residing in OAHs:

- Gender: Gender was found to be a significant predictor of depression. Compared to males (reference category), females had significantly higher odds of depression (OR = 123.85, 95% CI: 8.08–1898.44), indicating that female respondents were substantially more likely to experience depression.
- Type of illness: Type of illness was also significantly associated with depression. Using mental health problems as the reference category, respondents with physical health problems had significantly higher odds of depression (OR = 8.89, 95% CI: 1.33–59.20).
- Satisfaction with facilities and services in OAH: Regarding satisfaction with services and facilities in OAHs, respondents who reported a neutral level of satisfaction had significantly higher odds of depression compared to those who were very satisfied (OR = 211.88, 95% CI: 7.46–6014.55). However, no statistically significant associations were observed for those who were dissatisfied (*p* = 0.31) or satisfied (*p* = 0.09).

Overall, the findings indicate that gender, type of illness, and satisfaction with services and facilities in OAHs were significant predictors of depression among older adults. Females, individuals with physical health problems, and those reporting neutral satisfaction levels exhibited markedly higher odds of depression. In contrast, most sociodemographic, lifestyle and social support, health, and institutional (OAH)‐related variables were not statistically significant.

## 4 Discussion

The present study revealed that 82.3% of elderly residents in OAHs were at risk of depression, indicating a substantially high burden of psychological distress among institutionalized older adults. This prevalence is considerably higher than that reported in community‐based studies conducted in Bangladesh, which found prevalence rates 53% in urban and rural areas of Sylhet [52], 60% among older adults Dhamrai Upazila, Dhaka, and 59% in rural areas of Mirzapur, Tangail [53]. Beyond Bangladesh, prevalence rates of depression among older adults in India have shown considerable variation, ranging from 8.9% to 62.16% across different regional studies [34, 54, 55, 56] (Jammu and Sekhon). In European countries, studies have reported prevalence rates ranging from 18% to 37% [57]. Similarly, a cross‐sectional survey conducted in Korea found that 63% of the older adults experienced depression [58]. The observed variation in prevalence across studies may be attributed to differences in the theme of the research, size of sample, and sampling techniques, as well as variation in the measuring instruments.

The frequency of depression was shown to be significantly associated with sociodemographic, lifestyle and social support, health, and institutional (OAH)‐related factors, such as age, gender, occupation, children education levels, use of a personal mobile phone, current sickness, type of sickness, daily activities, type of OAHs, and services & facilities satisfaction offered by OAH authorities. These findings may support many other studies [10, 34, 53, 59, 60, 61, 62, 63]. Depression could be different according to gender. In this regard, the present study found that females had a higher prevalence of depression than males, and previous studies have found that women are more likely to be depressed than men [15, 34, 64, 65, 66, 67, 68, 69].

The present study found that older adults with physical health problems had significantly higher odds of depression compared to those with mental health problems. This aligns with previous studies showing that chronic physical conditions and functional limitations can reduce independence, increase pain, and negatively impact psychological well‐being, thereby elevating the risk of depressive symptoms [42, 70]. Institutionalized older adults often face multiple chronic conditions and physical limitations, which are important risk factors for depression, especially in low‐ and middle‐income countries [42]. Furthermore, physical frailty and limited functional ability in institutionalized older adults can heighten emotional distress and lower quality of life, increasing their risk of depression [71].

In this present study, we also found a significant association between depression and satisfaction with the services and facilities provided by the authorities in OAHs. These findings are consistent with national and international research demonstrating that the quality of services in institutional settings is closely linked to the psychological well‐being of older adults. Higher levels of depression among institutionalized elderly individuals in Bangladesh and other countries have been associated with limited recreational opportunities, inadequate caregiver support, insufficient medical care, and a lack of social engagement [30, 72, 73, 74, 75]. Due to restrictions on personal independence, adherence to a prescribed daily routine, and limited or no interaction with family, grandchildren, or their own children, elderly residents of OAHs may experience increased psychological stress and frustration even when institutional facilities are available. These factors can contribute to depression, which tends to be more severe among senior citizens. In governmental OAHs, where a large number of residents live in limited space, authorities are often able to provide only basic facilities, further exacerbating the residents' psychological distress.

In Bangladeshi society, elderly individuals who live with their families generally enjoy higher social status. Most families value and respect the experience and wisdom of older adults. Consequently, older people residing with their families tend to have a better quality of life and experience lower levels of depression compared to those living in institutional or assisted‐living settings [12, 76, 77, 78]. Previous studies have reported that elderly individuals living in OAHs face greater challenges compared to those residing with their families. These findings support earlier research indicating that older adults in institutional settings are more likely to experience psychological issues, including sadness, anxiety, loneliness, lack of family attention, and social isolation [79]. Similarly, multiple studies have shown that the overall rate of depression among older adults in OAHs was 81%, which is substantially higher than the 56.1% reported among those living in their own homes [80]. The present study found that 82.3% of older adults residing in OAHs were at risk of depression, consistent with findings from previous studies.

## 5 Conclusion

In our current study, we wanted to find out the sociodemographic, lifestyle and social support, health, and institutional (OAH)‐related factors that associate with depression and evaluate the overall risk of depression among the older people residing in OAHs. In general, the results indicate that the majority of elderly people reported experiencing depression. Getting older (above 60 years age), being female, having less service facilities, and having physical problems were found to be significant variables influencing risk of depression. Living alone and avoiding various social or physical activities also increases the depression risk. In our present study suggests that most of the elderly people spending their time just by walking, sleeping and doing nothing in OAHs. The study's results support the hypothesis that older people were more likely to experience depression. Our findings also suggest that in the OAH of Bangladesh, depression is common problem. It suggests that depression is turning into a silent killer that is spreading epidemically and reducing older people's quality of life. Therefore, additional consideration should be given to their healthcare and medical facilities, to ensure healthy and hygienic food for everyone. There also should be concerned to create a variety of recreational opportunities, to arrange different entertainment activities and to enhance their family relationships in order to raise their quality of life and mental health stability. Elderly people, especially females and those are ill, should be given extra attention for depression and treated them appropriately. The elderly should be included in paid or volunteer activities by the authorities. These findings may have a big influence on policy, especially when it comes to the welfare of those who are depressed, which involves detection at an early stage, engagement with society, and immediate rehabilitation. The government, non‐governmental organizations (NGO), different social welfare agencies/organizations, and health care departments should work together to develop a magnificent and executive plan for implementation that would improve geriatric health, particularly in the context of mental health.

### 5.1 Strengths and Limitations

In our study, we measured the depression level by using the internationally recognized Geriatric Depression Scale (GDS‐15, short version). Due to time constraints, lack of funds, and restricted access to the OAHs, we collected only 158 sample data. We visited many OAHs but found no residents there, some of them were closed and we had to leave. The authorities informed us that due to lack of sufficient funds, their OAHs are currently closed. Before collecting data in government OAHs, we had to apply for permission, which was time consuming and there were also some restrictions on accessibility and collecting information from the residents. Because of the health problems of residents, it was challenging to get accurate information. However, this study suggests a relationship but not causality because it is based on cross‐sectional information. Furthermore, additional clinical evaluation is necessary, as the Geriatric Depression Scale is a screening tool that only assesses the risk of depression.

## Author Contributions

**Mehedi Hasan Naim:** data curation, formal analysis, validation, writing – original draft. **Mimma Tabassum:** methodology, data curation, writing – review and editing, visualization, resources. **Md. Iftakhar Parvej:** conceptualization, data curation, supervision, project administration, writing – review and editing, funding acquisition, methodology, software, investigation.

## Conflicts of Interest

The authors declare no conflicts of interest.

## Transparency Statement

The lead author Md. Iftakhar Parvej affirms that this manuscript is an honest, accurate, and transparent account of the study being reported; that no important aspects of the study have been omitted; and that any discrepancies from the study as planned (and, if relevant, registered) have been explained.

## Data Availability

All relevant data are available on Kaggle repository through the link: https://www.kaggle.com/datasets/iftakhar021521/depression-oahs-in-bangladesh.
